# Comparing Balloon Dilation to Non-Balloon Dilation for Access in Ultrasound-Guided Percutaneous Nephrolithotomy: A Systematic Review and Meta-Analysis

**DOI:** 10.1590/S1677-5538.IBJU.2023.0373

**Published:** 2024-03-18

**Authors:** Meixuan Ding, Kai Zhu, Wenzhao Zhang, Haichao Huang, Bo Duan, Jiaxin Zheng, Huiqiang Wang, Tao Wang, Peide Bai, Chen Bin

**Affiliations:** 1 Fujian Medical University The School of Clinical Medicine Fuzhou China The School of Clinical Medicine, Fujian Medical University, Fuzhou, China; 2 Xiamen University School of Medicine The First Affiliated Hospital of Xiamen University Xiamen China The Key Laboratory of Urinary Tract Tumors and Calculi, Department of Urology Surgery, The First Affiliated Hospital of Xiamen University, School of Medicine, Xiamen University, Xiamen, China; 3 Xiamen University School of Medicine Women and Children's Hospital Xiamen China Department of Pediatric Surgery, Women and Children's Hospital, School of Medicine, Xiamen University, Xiamen, China

**Keywords:** Nephrolithotomy, Percutaneous, Kidney Calculi, Angioplasty, Balloon, Meta-Analysis [Publication Type]

## Abstract

**Purpose:**

This study aims to evaluate the safety and efficacy of ultrasound-guided balloon dilation compared to non-balloon dilation for percutaneous nephrolithotomy (PCNL).

**Materials and methods:**

A systematic review and meta-analysis were conducted by searching PubMed, EMBASE, and the Cochrane Library. Results were filtered using predefined inclusion and exclusion criteria as described and meta-analysis was performed using Review Manager 5.4 software.

**Results:**

A total of six studies involving 1189 patients who underwent PCNL were included. The meta-analysis results demonstrated that compared to non-balloon dilation, balloon dilation was associated with reduced haemoglobin drop [mean difference (MD) = -0.26, 95% CI = -0.40 ~ -0.12, P = 0.0002], decreased transfusion rate [odds ratio (OR) = 0.47, 95% CI = 0.24 ~ 0.92, P = 0.03], shorter tract establishment time (MD = -1.30, 95% CI = -1.87 ~ -0.72, P < 0.0001) and shorter operation time (MD = -5.23, 95% CI = -10.19 ~ -0.27, P = 0.04).

**Conclusions:**

Overall, ultrasound-guided balloon dilatation offered several advantages in PCNL procedures. It facilitated faster access establishment, as evidenced by shorter access creation time. Additionally, it reduced the risk of kidney injury by minimizing postoperative haemoglobin drop and decreasing the need for transfusions. Moreover, it enhanced the efficiency of surgery by reducing the operation time. However, it is important to note that the quality of some included studies was subpar, as they did not adequately control for confounding factors that may affect the outcomes. Therefore, further research is necessary to validate and strengthen these findings.

## INTRODUCTION

Percutaneous nephrolithotomy (PCNL) is a widely used treatment method for complex renal stones (1). Access dilation is a crucial step in PCNL and significantly affects the success of the procedure. Currently, the most common access dilation methods in PCNL include Amplatz dilation, Metal telescopic dilation, and Balloon dilation (2-4). In Europe and the United States, fluoroscopy is primarily utilized for access dilation during PCNL; it lacks the protection of radiation exposure but allows real-time monitoring of the tract establishment process (5, 6). Conversely, in China, ultrasound guidance is predominantly employed for access dilation (7-9).

Ultrasound-guided balloon dilation is becoming increasingly popular in Asian countries due to its real-time tract establishment monitoring and avoidance of radiation exposure. This technique has been widely accepted as safe and effective (10, 11). A previous meta-analysis has shown that fluoroscopically guided balloon dilation is safer and more effective than Amplatz dilation (4).

However, there are limited studies that focused on ultrasound-guided balloon dilation, and there is a lack of systematic reviews on this topic. Our hypothesis for this meta-analysis is that ultrasound-guided balloon dilation may simplify the surgical procedure, reduce access time, and lower the risk of bleeding-related complications. Therefore, the objective of this study is to analyse existing clinical evidence to compare the safety and efficacy of ultrasound-guided balloon dilation versus non-balloon dilation for tract dilation in PCNL. Additionally, we aim to investigate whether ultrasound-guided balloon dilation exhibits superior efficacy and safety compared to non-balloon dilation techniques.

## MATERIALS AND METHODS

This review is registered with PROSPERO (PROSPERO no. CRD 42023405292)

### Search strategy:

**Figure 1:**
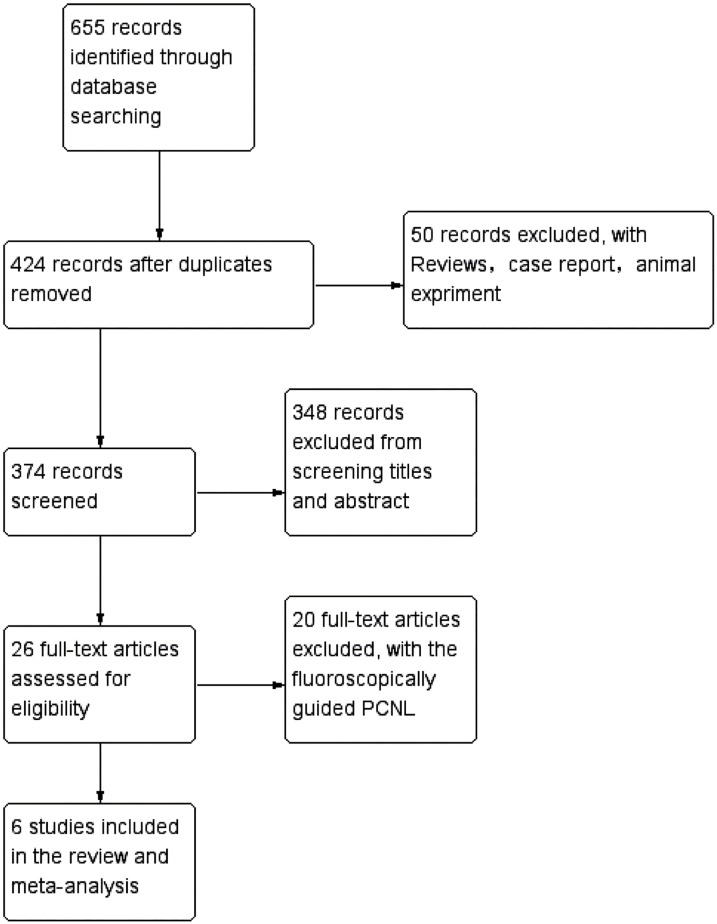
Flow diagram summarizing the process of literature selection.

Two reviewers conducted a systematic search of EMBASE, PubMed, and the Cochrane Library for relevant randomized controlled trials. Additionally, manual searching was performed to supplement the literature related to the included studies. The search was conducted up until October 11, 2022. The search terms used included Percutaneous Nephrolithotomy, PCNL, tract dilatation, access creation, balloon dilation and balloon dilator. The search strategy for each database was as follows: (“Nephrolithotomy, Percutaneous” [Mesh] OR “PCNL” OR “Nephrolithotomies, Percutaneous” OR “Percutaneous Nephrolithotomies” OR “Percutaneous Nephrolithotomy”) AND (“tract dilatation” [Mesh] OR “tract dilation” OR “access creation” OR “balloon dilation” OR “balloon dilator”). The search and selection process adhered to the requirements of the PRISMA guidelines. The specific details of the search and selection process for this study are presented in Figure-1.

### Inclusion and Exclusion criteria:

Randomized Controlled trials (RCTs) or Controlled Clinical trials (CCTs) comparing the clinical efficacy of balloon dilation versus non-balloon dilation for access creation in ultrasound-guided PCNL were included.

All patients were required to have no serious diseases before the operation, such as uncorrected anaemia or systemic bleeding disorders; severe heart disease and pulmonary insufficiency which would contraindicate surgery, and uncontrolled diabetes and/or hypertension. No studies were excluded based on these criteria.

The included studies reported at least one of the following outcomes: haemoglobin drop, transfusion rate, complication rate, successful dilation rate, stone-free rate, access time, total operation time and hospital stay. Studies with irregular endpoints were excluded.

Case reports, historical control studies, and reviews were also excluded.

Animal experiments were excluded from the study.

Grey literature such as meeting abstracts, posters etc. were excluded from the study.

### Data extraction and quality assessment:

Two authors extracted demographic characteristics and outcome data from studies according to the inclusion and exclusion criteria. The quality of the literature was assessed by reading the full text. The endpoints included haemoglobin drop, transfusion rate, complication rate, successful dilation rate, stone-free rate, access time, total operation time and hospital stay. Any discrepancies in data extraction were resolved through discussion with a third researcher.

The quality of the RCTs was evaluated using the Jadad scale, and the quality of the cohort and CCTs was assessed using the Newcastle-Ottawa scale. This assessment included factors such as appropriate randomization, sufficient allocation concealment, implementation of double-blinding, and instances of loss to follow-up, among others. According to the Jadad scale, studies scoring less than 3 are considered to be of low quality, whereas those with scores of 3 or higher are considered to be of high quality. Additionally, the Newcastle-Ottawa scale assigns scores ranging from one to nine, with research scoring less than 4 points are considered to be of low quality. The quality assessment of the included studies is presented in Table-1.

**Table 1:** Quality assessment of included studies.

NOS score	Selection	Comparability	Outcome	Score
Ren et al. 2014 (14)	★★	★★	★	5
Zhou et al. 2015 (15)	★	★	★★	4
Jin et al. 2020 (17)	★★★	★	★★	6
Tang et al. 2020 (18)	★★	★★	★	5
Wang et al. 2020 (19)	★★	★★	★★	6
Jadad score	Randomization	Double blinding	Withdrawals and dropouts	
Pakmanesh et al. 2019 (16)	2	2	1	5

### Data analysis:

The obtained literature was reviewed and the data was processed in accordance with the requirements of the Meta-analysis. Pooled effects were calculated using odds ratio (OR) with 95% confidence intervals (CIs) for dichotomous data, and mean difference (MD) with 95% CIs for continuous data. The statistical analysis was conducted using the random effects model (12). The heterogeneity of the data was assessed using I2 statistics and chi-square test. A larger I2 value indicates a higher level of heterogeneity. If necessary, sensitivity analysis was performed to determine the stability of the results. Review Manager 5.4 (Cochrane Collaboration, Copenhagen, Denmark) was utilized for the statistical analysis (13).

## RESULTS

### Search results:

A total of 655 relevant studies were initially identified, and after excluding those that did not meet the criteria, one RCT and five CCTs were included in the meta-analysis (Figure-1) (14-19). PCNL was conducted by experienced urologists in all patients. The baseline characteristics of the included studies are presented in Table-2.

**Table 2:** Baseline characteristics of balloon versus non-balloon for access dilation in PCNL.

Author	Year	Location	Types	Group	Sex (N, M/F)	Age (Years)	Stone burden	Access Sheath	PORS	NOS score
Ren et al. (14)	2014	China	CCT	BD	37/31	47.5±15.6	-	24F	-	5
				AMD	36/25	45.2±14.3	-	24F	-	
Zhou et al. (15)	2015	China	CCT	BD	18/29	48.8±13.0	2.4±0.6cm	22F	-	4
				AMD	16/29	49.6±12.9	2.3±0.6cm	22F	-	
Pakmanesh et al. (16)	2019	Iran	RCT	BD	17/16	47.21±17.13	578±448mm²	30F	7	5 a
				AMD	18/15	47.39±15.11	596±473mm²	30F	7	
Jin et al. (17)	2020	China	CCT	BD	-	-	-	30F	-	6
				TMD	-	-	-	24F	-	
Tang et al. (18)	2020	China	CCT	BD	35/33	53.04±13.58	2.91±0.59cm	24F	-	5
				AMD	18/8	53.15±11.11	2.56±0.61cm	16F	-	
Wang et al. (19)	2020	China	CCT	BD	115/92	51±10	3.6±1.2cm	24F	5	6
				SD	248/163	52±11	3.6±1.1cm	24F	11	

a Jadad scale score

RCT = Randomized controlled trials; CCT = Clinical controlled trials; BD = Balloon dilation; AMD = Amplatz dilation; TMD = Telescopic metal dilation; SD = Sequential dilation; PROS = Previous open renal surgery; NOS scale = Newcastle-Ottawa scale.

### Meta-analysis:

**Figure 2:**
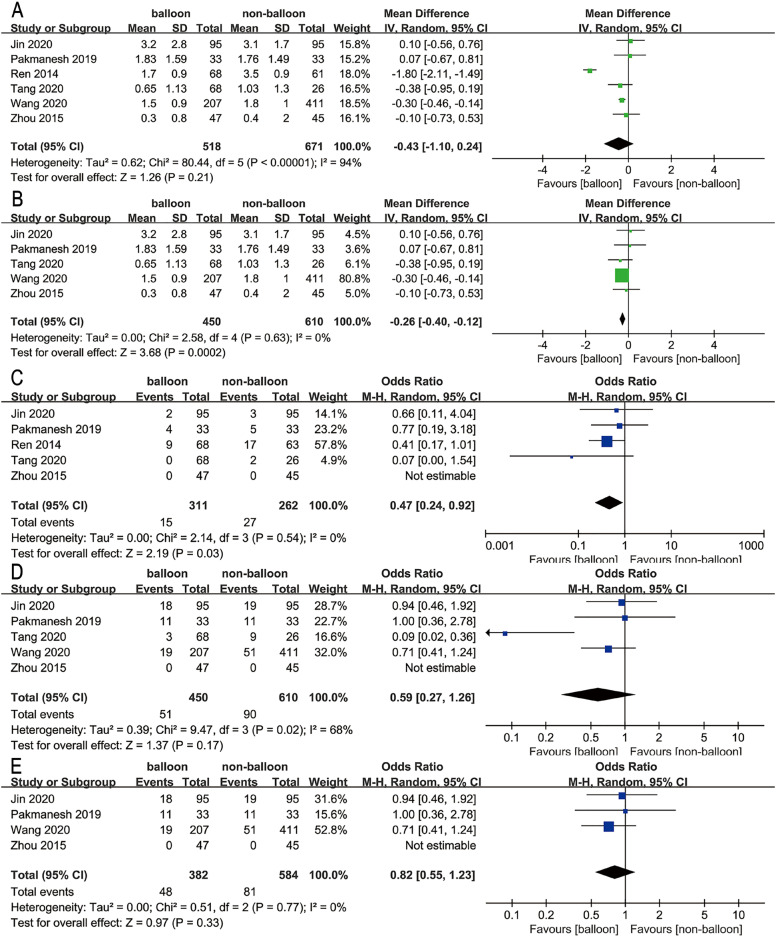
Forest plots showing the pooled results of (A) haemoglobin drop, (B) sensitivity analysis of haemoglobin drop, (C) transfusion rate, (D) complication rate, and (E) sensitivity analysis of complication rate.

**Haemoglobin drop:** Data on haemoglobin drop were provided by six of the included studies, involving 1,189 patients. Among these, two studies reported a significant reduction in haemoglobin drop with balloon dilation (14, 19), while the remaining four studies found no significant differences between balloon dilation and non-balloon dilation (15-18). The meta-analysis results suggest that there was no significant statistical difference in haemoglobin drop between the two groups (MD = -0.43, 95% CI = -1.10 ~ 0.24, P = 0.21). The heterogeneity test indicated that the heterogeneity between the studies was statistically significant (P < 0.00001, I2 = 94%) (Figure-2A).

To determine the source of this heterogeneity, we conducted a sensitivity analysis by excluding one study that had a substantial influence. After removing this study, there was no statistical significant heterogeneity between the remaining studies (P = 0.63, I2 = 0%). This variation may be attributed to the fact that Ren et al. study focused on patients with staghorn calculi. The results of the sensitivity analysis suggest that balloon dilation could effectively reduce haemoglobin drop (MD = -0.26, 95% CI = -0.40 ~ -0.12, P = 0.0002) (Figure-2B).

**Transfusion rate:** Five studies including 573 patients reported transfusion rates (14-18). Among them, one study concluded that balloon dilation significantly reduced the transfusion rate (14). The results of the meta-analysis indicated a statistically significant difference in transfusion rate between the two groups, with balloon dilation associated with a lower transfusion rate compared to non-balloon dilation (OR = 0.47, 95% CI = 0.24 ~ 0.92, P = 0.03). However, there was no significant heterogeneity observed (P = 0.54, I2 = 0%) (Figure-2C).

**Complication rate:** Among the included studies, five studies involving 1060 patients reported complication rates (15-19). However, none of these studies found that balloon dilation had a significant advantage in reducing the complication rate. The pooled analysis also did not show a significant difference in complication rates between the two groups (OR = 0.59, 95% CI = 0.27 ~ 1.26, P = 0.17). However, the results of the analysis were unstable and showed significant heterogeneity (P = 0.02, I2 = 68%) (Figure-2D).

After excluding one study that had a significant impact on heterogeneity, the heterogeneity between studies decreased (P = 0.77, I2 = 0%) (18). This could possibly be attributed to the long operative time and high intraoperative blood drop in the Tang et al. study. The results of the meta-analysis were consistent with previous studies, with no statistically significant difference in complication rates between the two groups (OR = 0.82, 95% CI = 0.55 ~ 1.23, P = 0.33) (Figure-2E).

**Figure 3:**
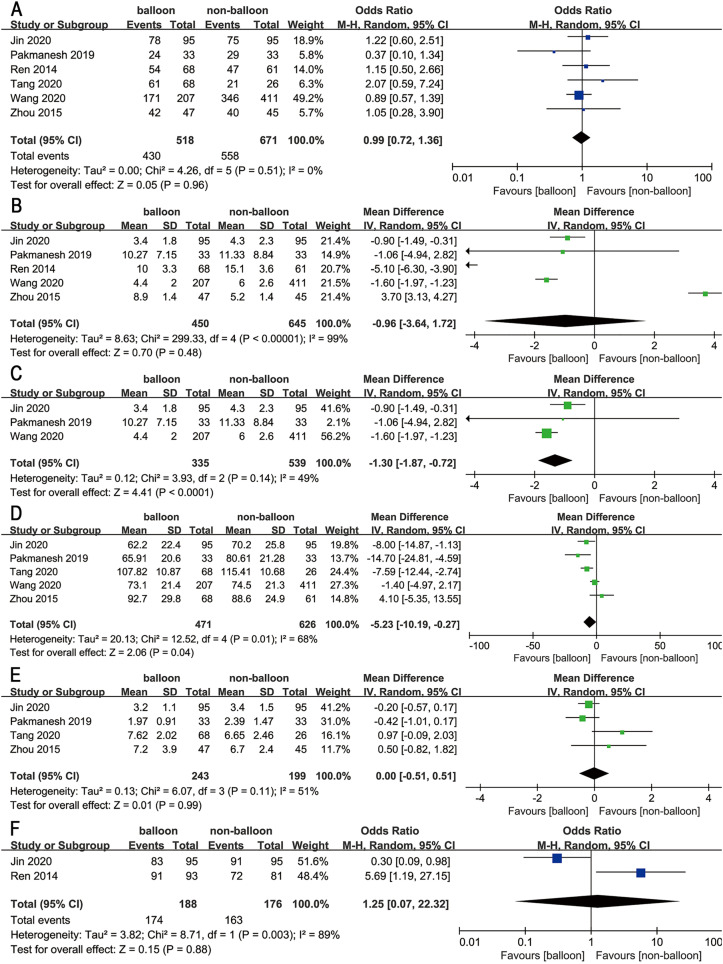
Forest plots showing the pooled results of (A) stone free rate, (B) tract establishment time, (C) sensitivity analysis of tract establishment time, (D) operation time, (E) hospital stay, and (F) successful dilation rate.

**Stone free rate:** Six studies involving 1189 patients reported the stone free rate, and none of these studies identified a significant difference in stone free rate between the two groups (14-19). No statistical significance of heterogeneity was observed among the studies (P = 0.51, I2 = 0%). The results of the meta-analysis indicated a slightly lower stone free rate in the balloon group compared to the non-balloon group, however, this difference was not statistically significant (OR = 0.99, 95% CI = 0.72 ~ 1.36, P = 0.96) (Figure-3A).

**Tract establishment time:** Five studies involving 1095 patients reported tract establishment time (14-17, 19), and three of them found that balloon dilation significantly reduced tract establishment time (14, 17, 19). However, Zhou et al. found that balloon dilation resulted in a longer tract establishment time compared to Amplatz dilation (15). The pooled effect showed no significant difference in tract establishment time between these two groups (MD = -0.96, 95% CI = -3.64 ~ 1.72, P = 0.48). Following a heterogeneity test, it was found that there was significant heterogeneity between studies (P < 0.00001, I2 = 99%) (Figure-3B).

After excluding two studies that had a greater impact on heterogeneity, the heterogeneity became acceptable (P = 0.14, I2 = 49%) (14, 15). The results of the meta-analysis showed that balloon dilation could significantly reduce the tract establishment time (MD = -1.30, 95% CI = -1.87 ~ -0.72, P < 0.0001) (Figure-3C).

**Operation time:** A total of five studies provided data on operative time (15-19), with three of them indicating that balloon dilation can significantly decrease the operation time (16-18). The findings of the meta-analysis also confirmed that there was a significant difference in the total operation time between the two groups. The pooled effect suggested that the operation time of the balloon dilation group was 5.23 minutes shorter than that of the non-balloon dilation group (MD = -5.23, 95% CI = -10.19 ~ -0.27, P = 0.04). However, there is substantial heterogeneity (I2 = 68%, P = 0.01) (Figure-3D).

**Hospital stay:** On the topic of hospital stay, four studies provided data. The heterogeneity was considered acceptable (P = 0.11, I2 = 51%) (15-18). The pooled effect indicated that the balloon group had a slightly longer hospital stay of 0.01 days compared to the non-balloon group. However, the results of the meta-analysis suggested that there was no significant statistical difference in hospital stay between the two groups (MD = 0.00, 95% CI = -0.51 ~ 0.51, P = 0.99) (Figure-3E).

**Successful dilation rate:** Two studies provided data on the successful dilation rate (14, 17). Ren et al. reported a significantly higher successful dilation rate with balloon dilation (14), while Jin et al. discovered that balloon dilation had a somewhat lower successful dilation rate compared to non-balloon dilation (17). The meta-analysis results showed that the successful dilation rate of balloon dilation was lower than that of non-balloon dilation, but the difference was not statistically significant (OR = 1.25, 95% CI = 0.07 ~ 22.32, P = 0.88), and there was substantial heterogeneity (P = 0.003, I2 = 89%). Due to the limited number of included studies, a sensitivity analysis was not conducted (Figure-3F).

## DISCUSSION

With the advancement of minimally invasive urological techniques, percutaneous nephrolithotomy (PCNL) has emerged as a preferred method for treating complicated kidney stones due to its benefits of minimal trauma, high stone clearance rate, and faster recovery (20, 21). In Europe and America, PCNL performed under X-ray guidance is considered the standard approach (10). However, Peng et al. found that fluoroscopically guided balloon dilation may offer certain advantages over Amplatz dilation (22). It is worth noting that X-ray-guided PCNL increases the risk of radiation exposure for doctors and patients (23). In recent years, the use of ultrasound guidance for PCNL has gained popularity in China (24). Previously, ultrasound-guided PCNL typically involved the use of Amplatz dilation or telescopic metal dilation, but these techniques lacked direct visualization and could potentially lead to renal injury. In contrast, recent reports have highlighted the use of balloon dilation, which can be monitored under ultrasound guidance. This approach simplifies the procedure, reduces access time, and lowers the risk of complications such as intraoperative bleeding (16, 17, 19). However, there is currently a lack of evidence-based medical data regarding the safety and efficacy of balloon dilation under ultrasound guidance. Therefore, this study aims to provide the first systematic review and meta-analysis of this technique.

Intraoperative blood drop and transfusion rate are important indicators for assessing the safety of surgery. The amount of haemoglobin drop indirectly reflects the extent of bleeding and blood loss during the procedure (25). The transfusion rate provides an objective measure of perioperative bleeding. Previous studies have shown that balloon dilation is a safer approach with fewer bleeding-related complications (8, 26, 27). Two of the studies included in this analysis demonstrated that balloon dilation significantly reduced haemoglobin drop (14, 19), and Ren et al. found that balloon dilation may also decrease transfusion rates (14). The results of the meta-analysis further support the notion that ultrasound-guided balloon dilation reduces bleeding and transfusion rates. Some researchers have reported that selecting the appropriate method for tract dilation and ensuring the correct puncture path can effectively prevent intraoperative bleeding (28). The advantages of ultrasound-guided balloon dilation during dilation and puncture may be attributed to the following factors: under ultrasound guidance, balloon dilation can avoid vascular injury by using Doppler flow image, and the balloon dilator expands radially and uniformly, exerting uniform pressure around the access site and compressing the adjacent small vessels, thereby reducing bleeding and minimizing intraoperative blood drop (29). Additionally, the balloon can be inflated to establish the standard channel in a single step, which shortens the procedure time and may further reduce the risk of intraoperative bleeding (18).

In this study, complications primarily included postoperative urinary tract infection, urinary leakage, bleeding, blood transfusion, postoperative fever, Double-J stent placement, and injury to the collecting system. However, there was inconsistency in the classification of complications among the included studies, which may have influenced the results of the statistical analysis. Sahan et al. (30) found that hydronephrosis grade, parenchymal thickness, duration of nephroscopy, and duration of nephrostomy c parenchyma during the dilation process (32, 33). Furthermore, performing balloon dilation under ultrasound guidance allows for direct visualization, reducing the risk of damage to the collecting system. Due to the inconsistent classification of complications among the studies, future studies should consider using a standardized approach to categorize various complications.

The successful dilation rate is a crucial endpoint for assessing the feasibility. Factors such as the patient's surgical history and the surgeon's experience significantly influence the rate of successful dilation. Skilled urologists with expertise in the field have reported a high rate of successful dilation due to their experience (8). On the other hand, Joel et al. found a failure rate of 25% for balloon dilation in patients with a history of open renal surgery (34). In our study, Ren et al. reported a significantly higher rate of successful dilation with balloon dilation in patients without a history of open renal surgery for staghorn stones (14). However, the meta-analysis conducted in this study did not show any significant differences in the successful dilation rat atheter were significantly associated with prolonged urinary leakage (30). Whether balloon dilation is effective in lowering complication rates remains controversial. Wang and Tang et al. found that ultrasound-guided balloon dilation was feasible and safe, with low complication rates compared to conventional gradual dilation, although the differences were not statistically significant (18, 19). Zhou et al. concluded that ultrasound-guided balloon dilation simplifies the access creation process, reduces the potential trauma associated with continuous operation of the Amplatz dilator, and results in fewer bleeding complications (15). Danilovic et al. reveals infundibula strictures can be found in 26.3% of the patients with residual stone fragments after standard PCNL for large burden kidney stones (31). This meta-analysis revealed a slightly lower complication rate in the balloon group (12.56%) compared to the non-balloon group (13.86%), although the difference was not statistically significant. This could be attributed to the specialized design tip of the access sheath of the balloon dilator, which causes less injury to the renal es between the two groups. It is important to note that only two studies reported successful dilation rates with high heterogeneity, therefore a sensitivity analysis was not performed. It is suggested that future studies should include this data for better evaluation.

The stone free rate is an important criterion for assessing effectiveness. According to the results from Tomaszewski et al., balloon dilation had a comparable stone free rate to non-balloon dilation in patients undergoing PCNL (35). Four studies included in our analysis reported slightly higher stone free rates in the balloon group, although no statistically significant difference was observed (14, 15, 17, 18). The results of the meta-analysis also demonstrated no significant difference in stone free rates between the two groups, which is consistent with the findings of the included studies. This suggests that there may not be a significant advantage of balloon dilation over non-balloon dilation in terms of stone free rates.

The time required for tract establishment is influenced by the complexity of the surgery and can impact the overall operation time. Three studies included in our analysis reported that balloon dilation significantly reduced the time required for tract establishment (14, 17, 19). Balloon dilation can be performed in a single step, without the need for sequential replacement of the access sheath. Additionally, the force exerted by the balloon on the tissue surrounding the tract is less likely to cause displacement, thereby preventing the loss of the access tract (33). However, Zhou et al. found that balloon dilation resulted in a longer time for tract establishment, but it did not have any impact on the total operation time (15). The meta-analysis revealed that the results regarding tract establishment time in the two studies were inconsistent (14, 15). After conducting a sensitivity analysis, it was found that balloon dilation significantly reduced the time required for tract establishment in patients without staghorn stones. This may be because the space between the renal calyx and the stone is often narrow in patients with staghorn calculi, leading to the guide wire sliding out or folding, resulting in loss of access.

The duration of the operation is associated with the risk of intraoperative infection and postoperative recovery. Several studies have found that the balloon group had significantly shorter operation times compared to the non-balloon group (16-18). This may be attributed to the use of softer and larger sheaths in balloon dilation. The use of softer sheaths allows for the extraction of larger stone fragments, reducing the time required for lithotripsy. Qin et al. meta-analysis, focusing on the treatment of renal stones larger than 2 cm, found that mini-PCNL did not demonstrate a significant advantage over the 24F standard-PCNL and, in fact, had a longer operation time (36). The results of the meta-analysis confirmed that balloon dilation significantly decreased the operation time, although there was substantial heterogeneity among the studies. Shortening the operation time can reduce the risks associated with anaesthesia and facilitate better patient recovery. Additionally, a shorter operation time can result in reduced bleeding, lower transfusion rates, and a decreased likelihood of infection by reducing the backflow of irrigation fluid into the bloodstream. However, with regard to hospital stay, the meta-analysis found no significant difference between the two groups.

This systematic review and meta-analysis have several limitations. Firstly, the number of included studies is relatively small, and most of them are retrospective studies with varying quality. Due to the limited number of relevant RCT studies, we combine RCT and CCT studies for analysis in our present study. Additionally, we conducted separate analyses for the CCTs and found that the results were generally consistent with our overall findings (data not shown). Nevertheless, these limitations may have impacted on the overall findings of our analysis. Secondly, due to the lack of available data, subgroup analysis based on factors such as BMI, stone burden, and previous history of open renal surgery was not performed to further explore the relationship between these factors and the choice of tract dilation technique. Thirdly, some outcome data in the included studies were incomplete and inconsistently reported, which may have introduced reporting bias. Lastly, the majority of the included studies are from Asia, which may introduce selection bias. Further research is required to validate the findings of this study.

## CONCLUSION

In conclusion, ultrasound-guided balloon dilation appears to be a safe and effective technique for dilation. It has the benefit of potentially reduce transfusion rates and haemoglobin drop, making it a potentially superior option. However, there was no significant difference in stone free rate and complication rate between the ultrasound-guided balloon dilation group and the control group. It is important to note that the number of studies and sample sizes included in this systematic review were relatively small, and many of them had limitations and biases. This aspect restricts the depth of our discussion and highlights the need for larger-scale, multi-centre, scientifically rigorous, and standardized randomized controlled trials to further validate the findings of this systematic review.

## Abbreviations

PCNL =: percutaneous nephrolithotomy
MD =: mean difference
OR =: odds ratio
RCTs =: Randomized controlled trials
CCTs =: Controlled Clinical Trials
